# Birthing parents had a lower risk of testing positive for SARS-CoV-2 in the peripartum period in Norway, 15^th^ of February 2020 to 15^th^ of May 2021

**DOI:** 10.1016/j.infpip.2021.100183

**Published:** 2021-11-10

**Authors:** Anders Skyrud Danielsen, Pascale-Renée Cyr, Maria Christine Magnus, Kirsten Midttun Gravningen, Hanne-Merete Eriksen-Volle, Oliver Kacelnik

**Affiliations:** a Department of Infection Control and Preparedness, Norwegian Institute of Public Health, Norway; b Cluster for Health Services Research, Norwegian Institute of Public Health, Norway; c Centre for Fertility and Health, Norwegian Institute of Public Health, Norway

**Keywords:** Covid-19, Pandemic, Preparedness, Peripartum care, Birth, Infection control

## Abstract

Hospital infection control measures against COVID-19 may come into conflict with patients' need for support. In Norway, some hospitals have restricted access for partners of women giving birth. We investigated the incidence rate of SARS-CoV-2 among birthing parents compared to similarly aged women and men in the general population; and the additional risk posed by allowing partners in. Birthing parents often shared infection status and had a slightly lower incidence rate than the general population in the peripartum period. They should not be considered a high-risk group for SARS-CoV-2 infections.

## Introduction

The need for strict infection control measures to protect both patients and staff during the COVID-19 pandemic has highlighted the conflict between restricting numbers of people entering hospitals against the need for emotional and physical support for patients. Throughout the pandemic, Norwegian hospitals have had different policies regarding whether partners should be allowed to accompany women giving birth, varying from following pre-pandemic practice, to only allowing partners in during the active stage of labour or refusing admittance completely [1]. Although the Norwegian Institute of Public Health (NIPH) has not explicitly defined pregnant women as a medical risk group, the institute has advised additional infection prevention measures for them.

We evaluated the SARS-CoV-2 transmission risk posed by birthing parents in delivery wards by assessing the incidence rate in peripartum mothers and their partners compared to the general population. We then assessed the frequency of concurrent infections among the parents and the number of pairs with discordant infection status.

## Methods

NIPH has a legal mandate to establish a national pandemic preparedness register (Beredt C19) [2]. The register contains data from national registers, including the National Population Register, the Medical Birth Registry (MBRN), the Norwegian Surveillance System for Communicable Diseases (MSIS), and the National Microbiology Laboratory Database. Data on individuals can be linked across registers by unique personal identification numbers. By law, all PCR-positive cases of SARS-CoV-2 must be reported to MSIS [3]. The NIPH has performed a data protection impact assessment for Beredt C19.

Using information from the MBRN, we identified all parents who had children between the 1^st^ of March 2020 to the 30^th^ of April 2021. These parents were “followed” throughout their peripartum period, which we defined as 14 days before and 14 days after delivery to account for infectiousness during delivery. We also selected a comparison group with an equal sex and age distribution from the general population who did not have a child during the pandemic. We assigned the comparison group a “pseudo delivery date” which was randomly drawn for each person from a monthly distribution that matched the parent group. We then followed the comparison group for 14 days before and after this date, as we did with the parents.

We explored the difference in incidence rate by fitting a median spline-smoothed line and a Cox regression model to the SARS-CoV-2 outcome by time in days. The hazard ratios (HR) of such a model can be interpreted as the estimated incidence rate ratio.

All analyses were performed using Stata SE 16.0 (College Station, TX, USA).

## Results

We included a total of 116,417 parents, 61,905 mothers and 54,512 partners (fathers/co-mothers). From the general population, we included 696,222 persons who did not have a child during the pandemic, of which 372,024 were females and 324,198 were males.

The incidence of SARS-CoV-2 was 0.17% (103/61,905) among the women giving birth and 0.15% (81/54,512) among the partners (Table S1). This was lower than the incidence in the general population, at 0.19% (695/372,024) for women and 0.18% (587/324,198) for men.

**Figure 1:**
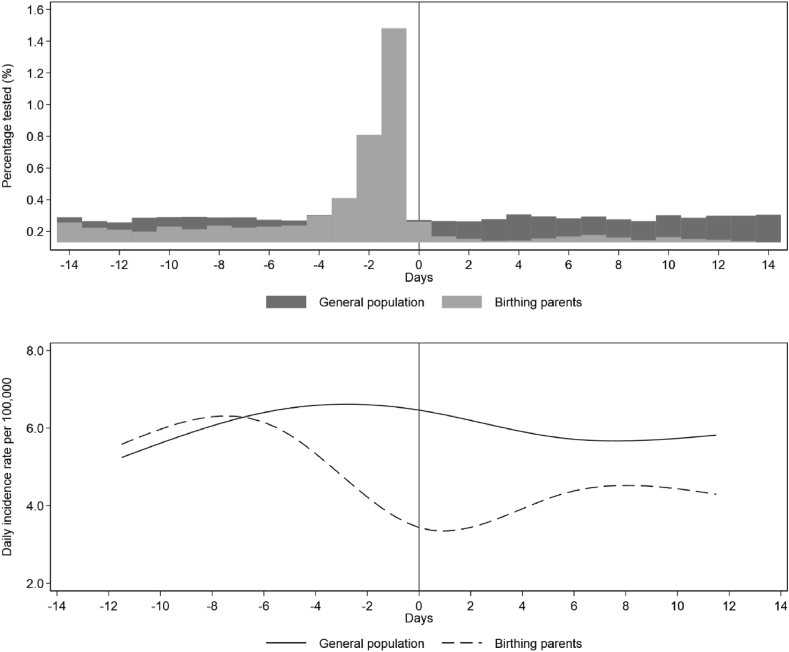
Upper panel is a bar plot displaying the percentage tested daily for cases and controls, and the lower panel is a median spline-smoothed line fit displaying the daily incidence rate per 100,000 for birthing parents and the general population during the peripartum period (pseudo birth date for the general population), 15th of February 2020 to 15th of May 2021, Norway.

The women giving birth were often tested one or two days before delivery (Figure 1). We also observed a higher incidence rate for birthing parents on these days, although the trend for both women giving birth and their partners was a lower overall incidence rate in the peripartum period.

An analysis of the infection status within couples showed that 51 (45%) of the couples had concurrent infections when excluding single parents (Table S2). In 35 (31%) of the couples, only the mother was infected, while in 28 (25%), only the partner was infected. Although we do not have data on whether the partner was present during delivery, of the 79 infected partners, 61 % (48/79) were infected before the delivery date, and likely not present.

The crude HR from the Cox regression model of being infected with SARS-CoV-2 in the peripartum period was 0.85 (0.72–0.99) for the birthing parents compared to the general population (Table 1). We also adjusted for three predictors of SARS-CoV-2 positivity in Norway – age, country of birth and urban residence – in addition to parity, which yielded an adjusted 0.84 (0.71–0.98). This corresponds to a 16% lower risk of being infected for the parents in this period. A post hoc test of the scaled Schoenfeld residuals indicated no violation of the proportional hazard assumption.

**Table I:** Hazard ratios of becoming SARS-CoV-2 positive during the peripartum period (pseudo birth date for the general population) from Cox regression models, 15th of February 2020 to 15th of May 2021, Norway. The study population is followed for 14 days before and 14 days after the date of delivery or the pseudo date of delivery. The adjusted model is adjusted for all listed covariates

	SARS-CoV-2 positivity			
	Crude		Adjusted	
	HR	95% CI	HR	95% CI
**Study groups**				
General population	1.00	-	1.00	-
Birthing parents	0.85	0.72–0.99	0.84	0.71–0.98
*Mothers*	*0.90*	*0.73–1.11*	*0.85*	*0.69–1.04*
*Partners*	*0.79*	*0.62–0.99*	*0.83*	*0.66–1.06*
**Age**				
Continuous	0.98	0.97–0.99	0.97	0.96–0.98
**Parity**				
0	1.00	-	1.00	-
1	1.07	0.94–1.22	1.24	1.09–1.42
2+	1.02	0.89–1.16	1.31	1.13–1.50
**Country of birth**				
Norway	1.00	-	1.00	-
High-income country	1.46	1.25–1.71	1.51	1.28–1.77
Low-/middle-income country	3.57	3.18–4.00	3.50	3.12–3.93
**Urbanicity**				
Rural municipality	1.00	-	1.00	-
Urban municipality	1.74	1.56–1.93	1.62	1.45–1.80

## Discussion

In this rapid policy-informing analysis, we found that mothers and their partners were slightly less often infected with SARS-CoV-2 during their peripartum period compared to the general population, despite the fact that they were tested more frequently as part of pre-triaging before delivery. Furthermore, we found that in couples registered with SARS-CoV-2 infection, both were often infected and thus presented a similar risk.

The increased incidence rate of mothers immediately prior to delivery can be due to pre-triage practice whereby high-risk individuals are identified and tested. The slightly lower incidence rate among birthing parents in the whole peripartum period, especially directly after delivery, may be because they increasingly shield themselves approaching their due date, perhaps due to fear of serious maternal disease [4].

The incidence of SARS-CoV-2 infection amongst expectant parents compared to the general population is not well documented, but a recent study from Norway found that pregnant women had a similar risk to non-pregnant women for testing positive during the entire pregnancy [5]. A study using surveillance data from UK, Sweden and USA also finds that birthing parents are not at an increased risk of a positive SARS-CoV-2 test despite more extensive testing [6]. Mexican surveillance data, however, shows a 15 % increased probability of test positivity among pregnant women compared to non-pregnant women [7]. A systematic review found that the prevalence of infection among pregnant women found through universal screening varied from 0% to 37% [8].

We found no study reporting the PCR positivity of partners of women giving birth or any assessment of the added risk they might contribute to the infection pressure in the hospitals. However, a study from Denmark reported partners more often had antibodies than the pregnant women themselves [9].

During the pandemic, NIPH emphasised that partners provide important support for women during delivery and their presence should be facilitated. Infection prevention measures should be appropriate, evidence-based, and proportional to the risk. This advice was based on the supposition that there was little additional risk posed to staff or other patients in having partners present, and that the measures taken to avoid infection, including adherence to pre-triage routines for screening expectant parents deemed at risk were sufficient. A study of the incidence of SARS-CoV-2 among health care workers in Norway did not indicate midwives were at a particular risk of contracting SARS-CoV-2 [10].

The completeness and quality of the pandemic preparedness register which allows us to follow the entire target population is an important strength of our analysis. The fact that Norway is a relatively low-prevalence setting, with a relatively small population that was followed for 15 months, may reduce generalisability. We have not been able to adjust for income and education, although some of these effects are captured by country of birth. Also, parents may have been infected just before or after our short study period window.

The implication of our study is that birthing parents, both mothers and their partners, should not be considered a high-risk population. The pandemic requires us to strengthen the infection prevention and control measures in hospitals, but where these measures are in place, ensuring expectant mothers receive the support they need should be paramount. The benefits of allowing partners to accompany delivering mothers is likely to outweigh the risk they pose.

## Credit author statement

**Anders Skyrud Danielsen**: Conceptualization, Data curation, Formal analysis, Investigation, Methodology, Writing – original draft, Writing – review & editing; **Pascale-Renée Cyr**: Data curation, Formal analysis, Investigation, Methodology, Writing – review & editing; **Maria Christine Magnus**: Data curation, Methodology, Writing – review & editing; **Kirsten Midttun Gravningen**: Writing – review & editing; **Hanne-Merete Eriksen-Volle**: Conceptualization, Project administration, Resources, Writing – review & editing; **Oliver Kacelnik**: Conceptualization, Project administration, Investigation, Methodology, Writing – original draft, Writing – review & editing.
