# Supplementary material for: Birthing parents had a lower risk of testing positive for SARS-CoV-2 in the peripartum period in Norway, 15th of February 2020 to 15th of May 2021
Source: Infect Prev Pract. 2021 Nov 10;3(4):100183. doi: 10.1016/j.infpip.2021.100183 (PMC8579713; doi:10.1016/j.infpip.2021.100183)
Supplement: Multimedia component 1 [file mmc1.docx]

# Supplementary material for *Birthing parents had a lower risk of testing positive for SARS-CoV-2 in the peripartum period in Norway, 15th of February 2020 to 15th of May 2021*

Table S1 - Description of the study population of birthing parents who was followed 14 days before and after the date of delivery, and the general population who was followed 14 days before and after an ascribed a uniformly distributed pseudo delivery date in the study period 15th of February 2020 to 15th of May 2021, Norway.

|  | **Birthing parents** | | | | | | **General population** | | | | | |
| --- | --- | --- | --- | --- | --- | --- | --- | --- | --- | --- | --- | --- |
|  | **Birthing parents in total** | | Mothers | | Partners | | **General population in total** | | Females | | Males | |
|  | **n** | **%** | n | % | n | % | **n** | **%** | n | % | n | % |
| **Total population** | **116417** | **-** | 61905 | - | 54512 | - | **696222** | **-** | 372024 | - | 324198 | - |
| **SARS-CoV-2 positivity** |  |  |  |  |  |  |  |  |  |  |  |  |
| Positive | 184 | 0.16 % | 103 | 0.17 % | 81 | 0.15 % | 1282 | 0.18 % | 695 | 0.19 % | 587 | 0.18 % |
| **Age** |  |  |  |  |  |  |  |  |  |  |  |  |
| <30 years | 35217 | 30.3 % | 22644 | 36.6 % | 12573 | 23.1 % | 210696 | 30.3 % | 135756 | 36.5 % | 74940 | 23.1 % |
| 30-39 years | 70421 | 60.5 % | 36479 | 58.9 % | 33942 | 62.3 % | 421164 | 60.5 % | 219294 | 58.9 % | 201870 | 62.3 % |
| >39 years | 10779 | 9.3 % | 2782 | 4.5 % | 7997 | 14.7 % | 64362 | 9.2 % | 16974 | 4.6 % | 47388 | 14.6 % |
| Age (median, interquartile range) | 32 (29-36) | | 31 (28-34) | | 33 (30-37) | | 32 (29-36) | | 31 (28-34) | | 33 (30-37) | |
| **Post-2008 parity** |  |  |  |  |  |  |  |  |  |  |  |  |
| 0 | 56829 | 48.8 % | 29134 | 47.1 % | 27695 | 50.8 % | 410244 | 58.9 % | 204754 | 55.0 % | 205490 | 63.4 % |
| 1 | 43225 | 37.1 % | 23720 | 38.3 % | 19505 | 35.8 % | 128833 | 18.5 % | 72246 | 19.4 % | 56587 | 17.5 % |
| 2+ | 16363 | 14.1 % | 9051 | 14.6 % | 7312 | 13.4 % | 157145 | 22.6 % | 95024 | 25.5 % | 62121 | 19.2 % |
| **Country of birth** |  |  |  |  |  |  |  |  |  |  |  |  |
| Norway | 84565 | 73.2 % | 44326 | 72.1 % | 40239 | 74.4 % | 503041 | 72.5 % | 270697 | 73.0 % | 232344 | 72.0 % |
| High-income country | 13667 | 11.8 % | 7306 | 11.9 % | 6361 | 11.8 % | 93328 | 13.5 % | 45236 | 12.2 % | 48092 | 14.9 % |
| Low-/middle-income country | 17348 | 15.0 % | 9879 | 16.1 % | 7469 | 13.8 % | 97039 | 14.0 % | 54815 | 14.8 % | 42224 | 13.1 % |
| *Missing information* | *837* |  | *394* |  | *443* |  | *2814* |  | *1276* |  | *1538* |  |
| **Urbanicity** |  |  |  |  |  |  |  |  |  |  |  |  |
| Urban municipality | 33552 | 28.8 % | 17921 | 28.9 % | 15631 | 28.7 % | 224511 | 32.2 % | 122663 | 33.0 % | 101848 | 31.4 % |

Table 2 - Concurrence and non-concurrence in SARS-CoV-2 positivity between parents who were couples (single parents excluded) during the peripartum period, 15th of February 2020 to 15th of May 2021, Norway, a total of 114 couples with any infection and 165 infected non-single parents.

| **Pairs** | **Frequency** |
| --- | --- |
| Pairs where only the mother is infected | 35 |
| Pairs where only the partner is infected | 28 |
| Pairs where both are infected | 51 |
